# Supplementary material for: Zooplankton-phytoplankton biomass and diversity relationships in the Great Lakes
Source: PLoS One. 2023 Oct 26;18(10):e0292988. doi: 10.1371/journal.pone.0292988 (PMC10602272; doi:10.1371/journal.pone.0292988)
Supplement: Attachment — Submitted filename: PONE-D-23-03077_reviewer_comments.pdf [file pone.0292988.s012.pdf]

PLOS ONE

Zooplankton-phytoplankton biomass and diversity relationships in the Great Lakes

--Manuscript Draft--

**Manuscript Number:**	PONE-D-23-03077
**Article Type:**	Research Article
**Full Title:**	Zooplankton-phytoplankton biomass and diversity relationships in the Great Lakes
**Short Title:**	Zooplankton-phytoplankton relationships in the Great Lakes
**Corresponding Author:**	Katya Kovalenko<br>University of Minnesota Duluth<br>Duluth, MN UNITED STATES
**Keywords:**	
**Abstract:**	Quantifying the relationship between phytoplankton and zooplankton may offer insight into zooplankton sensitivity to shifting phytoplankton assemblages and the potential impacts of producer-consumer decoupling on the rest of the food web. We analyzed 18 years (2001 – 2018) of paired phytoplankton and zooplankton samples collected as part of US EPA's Great Lakes Biological Monitoring Program to examine both the long-term and seasonal relationships between zooplankton and phytoplankton across all five Laurentian Great Lakes. We also analyzed effects of phytoplankton diversity on zooplankton biomass, diversity, and predator-prey (zooplanktivore/grazer) ratios. Across the Great Lakes, there was a weak positive correlation between total algal biovolume and zooplankton biomass in both spring and summer. The relationship was weaker and not consistently positive within individual lakes. These trends were consistent over time, providing limited evidence of increasing decoupling over the study period. Zooplankton biomass was weakly negatively correlated with algal diversity across lakes, whereas zooplankton diversity was unaffected. These relationships did not change when we considered only the edible phytoplankton fraction, possibly due to the high correlation between total and edible phytoplankton biovolume in most of these lakes. Lack of strong coupling between these producer and consumer assemblages may be related to lagging responses by the consumers, top-down effects from higher-level consumers, or other confounding factors. These results underscore the difficulty in predicting higher trophic level responses, including zooplankton, from changes in phytoplankton assemblages.
**Order of Authors:**	Katya Kovalenko
	Euan D. Reavie
	Stephanie Figary
	Lars G. Rudstam
	James M. Watkins
	Anne Scofield
	Christopher T. Filstrup
**Additional Information:**	
**Question**	**Response**
**Financial Disclosure**<br><br>Enter a financial disclosure statement that describes the sources of funding for the work included in this submission. Review the submission guidelines for detailed requirements. View published research articles from *PLOS ONE* for specific examples.	This study was funded by the U.S. Environmental Protection Agency under Cooperative Agreements GL-00E23101-2 and GL-00E0198-0 (ER).<br><br>https://www.epa.gov/aboutepa/about-great-lakes-national-program-office-glnpo<br><br>The funders had no role in study design, data collection and analysis, decision to publish, or preparation of the manuscript.

Powered by Editorial Manager® and ProduXion Manager® from Aries Systems Corporation

This statement is required for submission and **will appear in the published article** if the submission is accepted. Please make sure it is accurate.<br><br>**Unfunded studies**<br>Enter: *The author(s) received no specific funding for this work.*<br><br>**Funded studies**<br>Enter a statement with the following details:<br>• Initials of the authors who received each award<br>• Grant numbers awarded to each author<br>• The full name of each funder<br>• URL of each funder website<br>• Did the sponsors or funders play any role in the study design, data collection and analysis, decision to publish, or preparation of the manuscript?<br>• **NO** - Include this sentence at the end of your statement: *The funders had no role in study design, data collection and analysis, decision to publish, or preparation of the manuscript.*<br>• **YES** - Specify the role(s) played.<br><br>* typeset	
**Competing Interests**<br><br>Use the instructions below to enter a competing interest statement for this submission. On behalf of all authors, disclose any competing interests that could be perceived to bias this work—acknowledging all financial support and any other relevant financial or non-financial competing interests.<br><br>This statement is required for submission and **will appear in the published article** if the submission is accepted. Please make sure it is accurate and that any funding sources listed in your Funding Information later in the submission form are also declared in your Financial Disclosure statement.<br><br>View published research articles from	The authors have declared that no competing interests exist.

PLOS ONE for specific examples.<br><br>**NO authors have competing interests**<br><br>Enter: *The authors have declared that no competing interests exist.*<br><br>**Authors with competing interests**<br><br>Enter competing interest details beginning with this statement:<br><br>*I have read the journal's policy and the authors of this manuscript have the following competing interests: [insert competing interests here]*<br><br>* typeset	
**Ethics Statement**<br><br>Enter an ethics statement for this submission. This statement is required if the study involved:<br><br>• Human participants<br>• Human specimens or tissue<br>• Vertebrate animals or cephalopods<br>• Vertebrate embryos or tissues<br>• Field research<br><br>Write "N/A" if the submission does not require an ethics statement.<br><br>General guidance is provided below. Consult the submission guidelines for detailed instructions. **Make sure that all information entered here is included in the Methods section of the manuscript.**	NA this submission is using existing (field) data

Powered by Editorial Manager® and ProduXion Manager® from Aries Systems Corporation

**Format for specific study types**<br><br>**Human Subject Research (involving human participants and/or tissue)**<br>• Give the name of the institutional review board or ethics committee that approved the study<br>• Include the approval number and/or a statement indicating approval of this research<br>• Indicate the form of consent obtained (written/oral) or the reason that consent was not obtained (e.g. the data were analyzed anonymously)<br><br>**Animal Research (involving vertebrate animals, embryos or tissues)**<br>• Provide the name of the Institutional Animal Care and Use Committee (IACUC) or other relevant ethics board that reviewed the study protocol, and indicate whether they approved this research or granted a formal waiver of ethical approval<br>• Include an approval number if one was obtained<br>• If the study involved *non-human primates*, add *additional details* about animal welfare and steps taken to ameliorate suffering<br>• If anesthesia, euthanasia, or any kind of animal sacrifice is part of the study, include briefly which substances and/or methods were applied<br><br>**Field Research**<br><br>Include the following details if this study involves the collection of plant, animal, or other materials from a natural setting:<br>• Field permit number<br>• Name of the institution or relevant body that granted permission	
**Data Availability**<br><br>Authors are required to make all data underlying the findings described fully available, without restriction, and from the time of publication. PLOS allows rare exceptions to address legal and ethical concerns. See the PLOS Data Policy and FAQ for detailed information.	Yes - all data are fully available without restriction

A Data Availability Statement describing where the data can be found is required at submission. Your answers to this question constitute the Data Availability Statement and **will be published in the article**, if accepted.<br><br>**Important:** Stating 'data available on request from the author' is not sufficient. If your data are only available upon request, select 'No' for the first question and explain your exceptional situation in the text box.<br><br>Do the authors confirm that all data underlying the findings described in their manuscript are fully available without restriction?	
**Describe where the data may be found in full sentences. If you are copying our sample text, replace any instances of XXX with the appropriate details.**<br><br>• If the data are **held or will be held in a public repository**, include URLs, accession numbers or DOIs. If this information will only be available after acceptance, indicate this by ticking the box below. For example: *All XXX files are available from the XXX database (accession number(s) XXX, XXX.*).<br>• If the data are all contained **within the manuscript and/or Supporting Information files**, enter the following: *All relevant data are within the manuscript and its Supporting Information files.*<br>• If neither of these applies but you are able to provide **details of access elsewhere**, with or without limitations, please do so. For example:<br><br>*Data cannot be shared publicly because of [XXX]. Data are available from the XXX Institutional Data Access / Ethics Committee (contact via XXX) for researchers who meet the criteria for access to confidential data.*<br><br>*The data underlying the results presented in the study are available from (include the name of the third party*	will be held in a public repository<br>Some relevant data are within the manuscript and its Supporting Information files.

and contact information or URL). • This text is appropriate if the data are owned by a third party and authors do not have permission to share the data. <br>* typeset	
Additional data availability information:	Tick here if the URLs/accession numbers/DOIs will be available only after acceptance of the manuscript for publication so that we can ensure their inclusion before publication.

Powered by Editorial Manager® and ProduXion Manager® from Aries Systems Corporation

Click here to access/download;Manuscript;zooPhyto_plos_one.docx

Zooplankton-phytoplankton biomass and diversity relationships in the Great Lakes

Katya E. Kovalenko [a,d], Euan D. Reavie [a], Stephanie Figary [b],

Lars G. Rudstam[b], James M. Watkins [b], Anne Scofield [c], Christopher T. Filstrup [a]

[a] Natural Resources Research Institute, University of Minnesota, Duluth, USA

[b] Department of Natural Resources and Cornell Biological Field Station, Cornell University, Ithaca, USA

[c] EPA Great Lakes National Program Office, Chicago, IL, USA

[d] philarctus@gmail.com

Abstract

Quantifying the relationship between phytoplankton and zooplankton may offer insight into zooplankton sensitivity to shifting phytoplankton assemblages and the potential impacts of producer-consumer decoupling on the rest of the food web. We analyzed 18 years (2001 – 2018) of paired phytoplankton and zooplankton samples collected as part of US EPA's Great Lakes Biological Monitoring Program to examine both the long-term and seasonal relationships between zooplankton and phytoplankton across all five Laurentian Great Lakes. We also analyzed effects of phytoplankton diversity on zooplankton biomass, diversity, and predator-prey (zooplanktivore/grazer) ratios. Across the Great Lakes, there was a weak positive correlation between total algal biovolume and zooplankton biomass in both spring and summer. The relationship was weaker and not consistently positive within individual lakes. These trends were consistent over time, providing limited evidence of increasing decoupling over the study period. Zooplankton biomass was weakly negatively correlated with algal diversity across lakes, whereas zooplankton diversity was unaffected. These relationships did not change when we considered only the edible phytoplankton fraction, possibly due to the high correlation between total and edible phytoplankton biovolume in most of these lakes. Lack of strong coupling between these producer and consumer assemblages may be related to lagging responses by the consumers, top-down effects from higher-level consumers, or other confounding factors. These results

underscore the difficulty in predicting higher trophic level responses, including zooplankton, from changes in phytoplankton assemblages.

Introduction

With a few rare exceptions, aquatic ecosystems in the Anthropocene have experienced changes in temperature and nutrient concentrations, which can lead to shifts in phytoplankton assemblages [1-3]. In many cases, these compositional changes can alter the seasonal timing and amplitude of primary productivity [4,5] and functional attributes of phytoplankton [6,7]. Changes in phytoplankton assemblage composition and dynamics can lead to decoupling of primary producers and consumers, which may destabilize planktonic food webs with cascading effects on tertiary consumers [8-10].

Theory predicts and observational studies have shown that greater phytoplankton diversity is linked to increased phytoplankton resource use efficiency (horizontal diversity effects within trophic levels, [11]) and to increased zooplankton growth rate, diversity, and abundance (vertical diversity effects across trophic levels [12]). Phytoplankton diversity can also directly influence consumers via biochemical diversity in food resources which should increase zooplankton diversity [13] and these diversity effects may produce direct and indirect feedbacks to buffer primary consumer populations and entire food webs from abrupt shifts in their resource base. Because phytoplankton diversity can decrease variability in zooplankton productivity [12], greater algal diversity may support more zooplankton predators and therefore greater predator-prey ratios within the zooplankton community. However, diversity effects are not consistent across systems [14] and different measures of phytoplankton diversity can have opposing influences on horizontal and vertical diversity effects [15]. For example, communities dominated by cyanobacteria have a larger proportions of inedible taxa [16], which may limit zooplankton biomass [17,18] or have no impact [19].

The structure of large lake food webs is less understood than that of smaller lakes [20,21], and previous vertical diversity studies have largely focused on smaller ecosystems. In the Laurentian Great Lakes, several attributes of phytoplankton assemblages, including total biovolume, cell densities, average cell sizes, and species composition, have fluctuated considerably in the last few decades, with likely causes being changes in nutrient availability, invasive species, and climate change [5,22,23,24]. At the same time, zooplankton shifted to greater dominance by calanoid copepods, particularly *Limnocalanus macrurus* [25], increased abundances of the predatory invasive cladoceran *Bythotrephes* in some lakes [26], and changes in zooplankton vertical distribution with migrations to greater depths as an anti-predatory response to *Bythotrephes* [27]. With a wealth of long-term historical data, there have been multiple detailed analyses of trends in specific assemblages [22,28] and concurrent trends [29,30]; however, the degree of zooplankton and phytoplankton coupling, vertical diversity effects, and detailed associations between specific groups of taxa are less well understood.

Ideally, investigations of the relationships between primary producers and consumers should use high-resolution productivity data and information on feeding selectivity [31]. However, long-term high-resolution *in situ* productivity data are relatively sparse and often limited to smaller geographic areas (e.g., [32]), and landscape-scale analyses often rely on standing biomass. Controlled studies of feeding selectivity, usually conducted in laboratory settings, are similarly difficult to extrapolate to diverse and dynamic natural settings. We used nearly 20 years of paired zooplankton and phytoplankton data from the US EPA Great Lakes Biological Monitoring Program to examine ecological associations, long-term and seasonal dynamics of zooplankton-phytoplankton coupling, and effects of phytoplankton diversity on zooplankton biomass and diversity. We predicted that there would be a positive correlation between algal biovolume and zooplankton biomass, and that the slope of this relationship would decrease over time as a result of increasing decoupling of the two trophic levels associated with changes in phytoplankton assemblages. We also tested relationships between algal diversity and total

zooplankton biomass, zooplankton diversity, and zooplanktivore-grazer ratios, and explored group-level associations between the major types of zooplankton and algae.

Materials and Methods

We used data collected as part of the US EPA Great Lakes Biology and Water Quality Monitoring Programs in the pelagic Laurentian Great Lakes of North America, focusing on years which had matching phytoplankton and zooplankton data (2001 – 2018). Samples are collected twice a year in the spring (usually April) and summer (usually August) from 72 sites across the five Great Lakes: Lakes Erie, Ontario, Huron, Michigan, and Superior (SI Table 1). For phytoplankton, equal volumes of water were collected by a rosette sampler from multiple depths (0, 5, 10, 20 m) at each station representing the upper 20 m of the isothermal water column in the spring or the epilimnion in the summer [22]. Four spring samples from individual depths were composited to form an integrated sample; in summer, a minimum of two and maximum of four depths (typically 0, 5, 10 m, and lower epilimnion, but fewer taken when the mixed layer is shallow) were composited to form a representative sample from the epilimnion [33]. Samples were preserved with Lugol's iodine solution and analyzed as described in EPA GLNPO standard operating procedure [34]. We used the Utermöhl method [35] for soft-bodied algal identification. Subsamples were processed for detailed diatom assessment by acid digestion, slide-mounting and high-resolution microscopy. Algal specimens were also measured to allow for biovolume calculations [36].

Phytoplankton taxa were characterized as edible or inedible based on a combination of entity shape and nutritional quality. Characterization of edibility in freshwater phytoplankton has been considered previously [37], and we followed similar methods. We assumed that cyanobacteria are less desirable food organisms due to their poor nutritional quality [38]. Further, we considered a prevailing size and shape of entities (as single cells, filaments, globular colonies) greater than 50 µm to be inedible.

Therefore, algae such as filamentous diatoms are considered problematic as food for zooplankton despite their high nutritional value. We acknowledge that previously published assumptions around edibility are overly simplistic, and that edibility of a given phytoplankton taxon is likely grazer-specific. For instance, some larger zooplankton taxa may be equipped to disaggregate large, filamentous diatoms into edible sizes, as noted in a limited set of species-specific studies from marine systems (e.g., [39]). Such nuances should be considered in the future, but we treat our analyses as a first attempt to evaluate this phenomenon in the Great Lakes. Using these edibility criteria, we filtered out all phytoplankton taxa with low nutritional and low shape edibility (SI Table 2), and recalculated biovolume of remaining phytoplankton at each site.

Crustacean zooplankton, dreissenid veligers, and rotifers were collected by vertical tows taken across the same depth range, at the same time and stations as the phytoplankton data. All samples were collected according to EPA GLNPO standard operating procedure LG402 [40] and analyzed following LG403 [41]. Samples used here were collected using a 63 µm mesh net towed from 20 m or 1 m above the bottom, whichever was shallower, to the surface, at a rate of 0.5 m/s. As with phytoplankton, zooplankton sample collection for this program occurs 24 hours a day, and some stations are sampled during the day and some at night. Zooplankton samples from 20 m were not available for 2007 (both seasons) and for the spring season 2008-2011. Plankton were narcotized with soda water and preserved with sucrose formalin. Separate counts with different subsampling approaches were done for crustaceans and microzooplankton (rotifers, veligers, nauplii) and data combined to densities (numbers/ m^3). A minimum of 400 individuals for each of the two counts were identified to the smallest practical taxonomic unit (moslty species) and up to 20 individuals in each taxonomic unit were measured for length in mm using computerized [41]. Dry weight individual biomass (µg) was calculated from taxa-specific length-weight regressions available in the standard operating procedures [25,41]. Some rotifer equations used width measurements.

Statistical analyses

We used simple linear models to test for correlations between phytoplankton biovolume and zooplankton biomass, and correlations between phytoplankton and zooplankton diversity. All biovolume analyses were repeated with total and edible phytoplankton biovolume. Data distribution was checked using *qqnorm* function in R and log-transformation was applied to reduce skewness when warranted. Additionally, cross-correlation analyses were used to visualize the relationships between key groups of phytoplankton and zooplankton (Table S2). Zooplankton predator ratios were calculated using the sum of predatory cladocerans (*Bythotrephes, Cercopagis, Leptodora* and *Polyphemus*) and *Limnocalanus* biomass relative to other zooplankton, although the latter varies in its degree of zooplanktivory across the Great Lakes [42]. All analyses were done in R [43].

Results

Across the 20 years of data and all of the lakes, there was a weak positive correlation between total algal biovolume and zooplankton biomass ($P < 0.0001$, $R^2 = 0.21$). This Great Lakes-wide correlation was season-dependent, with the overall trend driven primarily by the summer ($P < 0.0001$, $R^2 = 0.22$, vs. spring $R^2 = 0.06$, Fig. 1). The relationship was also scale-dependent and varied across individual lakes, with a positive relationship in Lake Erie in both seasons and in Lake Huron in the spring, but a lack of significant correlations in lakes Ontario, Superior and Michigan in either season (Fig. 1). The slopes of biomass-biovolume relationship (evidence of decoupling) did not change uniformly with time ($P > 0.05$, Fig. 2).

Fig. 1. Phytoplankton biovolume and zooplankton dry biomass correlations for all lakes and for individual Laurentian Great Lakes by season.

Fig. 2. Temporal dynamics of the phytoplankton-zooplankton relationship. Fewer stations had matching data for the two assemblages earlier in the time series and spring data was unavailable for zooplankton between 2008 – 2011 (see Table S1 for complete summary of stations sampled by year).

Total zooplankton biomass was weakly negatively correlated with phytoplankton Shannon diversity ($P = 0.001$, $R^2 = 0.04$) and this relationship was relatively constant for both spring and summer across all lakes (Fig. 3). This correlation was driven largely by Lake Erie, which spanned the longest gradient of both biomass and diversity, and was less pronounced in other lakes. Zooplankton diversity was not closely associated with phytoplankton diversity ($R^2 < 0.02$, Fig. S1). Most of the biomass of different zooplankton groups was unrelated or weakly negatively related to overall algal richness and diversity, with the exception of a stronger positive relationship for *Limnocalanus* ($R^2 = 0.26$, Fig. 4). The majority of zooplankton groups had closer associations with other zooplankton groups, followed by biovolumes of Cyanophyta, Chlorophyta, and total algal biovolume (Fig. 4). Some variation in zooplankton predator-prey ratios was explained by algal diversity ($P < 0.0001$), whereas algal richness and biovolume did not have a strong linear effect (Fig. S2).

Fig. 3. Total zooplankton biomass as a function of phytoplankton diversity (Shannon H) across all lakes and for individual Great Lakes by season.

Fig. 4. Cross-correlation matrix for key groups of zooplankton and phytoplankton (Spearman correlation coefficients color-coded by shade intensity); all biovolume and biomass metrics have been log10-transformed. Relationships with visible R have $P < 0.0001$.

Edible algal biovolume was closely correlated with the overall algal biovolume (across lakes $R^2 = 0.78$, $P < 0.0001$ in each lake), with the largest discrepancy observed for Lake Erie (Fig. 5), where cyanobacteria are abundant in the summer. Our edibility criteria excluded algae with low nutritional value as well as those with difficult to manipulate shapes; we did not consider the two types of edibility filters separately, because even in the extreme scenario, there was a close relationship with total algal biovolume. Because of this relatively high correlation, most of the zooplankton-phytoplankton relationships were not greatly affected when considering only edible phytoplankton biovolume (Figs. S3, S4). In most cases, time of day had minimal effect on the zooplankton-phytoplankton relationship (Fig. S5). The relationship between total and edible biovolume did not exhibit directional changes over time (Fig. S6).

Fig. 5. Relationship between edible and total phytoplankton biovolume across the Great Lakes. White line indicates the 1:1 ratio, the degree of departure from this line illustrates decreasing relative biovolume of edible algal taxa.

Discussion

There was a significant correlation between phytoplankton biovolume and zooplankton biomass across this long-term, large-scale dataset; however, it only held across the entire basin, and not individual lakes, and only in the summer. The weak correlation between phytoplankton biovolume and zooplankton biomass could result from a lag in the response of zooplankton consumers to algal changes or variable top-down forcing on zooplankton across the lakes. If a lag in consumer response is present, we would expect the relationship to be stronger in the summer, which was generally the case, even though the correlation was still very poor in terms of predictive power and mostly insignificant for individual lakes. It is not surprising that the large trophic gradient of those lakes, from oligotrophic to

meso-eutrophic, was also reflected by the gradient in zooplankton biomass and phytoplankton biovolume across the entire basin. Similarly, in other lakes the coupling between phytoplankton biomass and zooplankton biomass was limited beyond a certain productivity level [44, 45 for Lakes Balaton and Lake Constance].

The slope and strength of the relationship between phytoplankton and zooplankton did not vary significantly with time, despite considerable shifts in algal and zooplankton community composition and productivity [5,22,25], providing little additional evidence for a disruption in coupling of producers and consumers. Size spectra slopes across picoplankton to macrozooplankton in this system have also been relatively stable over this time period [46], which may also explain lack of changes in coupling.

The relationships of zooplankton biomass and diversity with edible phytoplankton were similar to those with total phytoplankton biovolume, likely because edible and total phytoplankton biovolume were closely correlated in all lakes with exception of Lake Erie, the most productive lake with a greater incidence of harmful algal blooms. Although other studies have shown that although the proportion of inedible phytoplankton, particularly Cyanobacteria, increases in higher productivity lakes [16,47,48], cyanobacteria can also be abundant in oligotrophic systems [49] and can represent large proportions of biomass across large total phosphorus gradients [50]. Increasing biomass of less-edible phytoplankton, such as Cyanobacteria, has been observed to limit zooplankton resource use efficiency and the structure of trophic interactions [16]. However, the relationship between cyanobacterial blooms and zooplankton is variable, and previous studies have observed positive correlations between harmful algal blooms and several groups of zooplankton [19].

Bottom-up forcing was demonstrated to be important in Lakes Michigan and Huron [46], where declines in zooplankton biomass and particularly grazer cladocerans were associated with simultaneous declines in spring chlorophyll indicating potential grazer limitation [29,51,52]. In other cases, changes in zooplankton are better explained by top-down forcing through increased invertebrate or fish predation

[26,32,53], including changes in vertical distribution [54]. It is likely that the relative importance of these forces varies across the large spatial and trophic gradient and with season, contributing to the overall uncertainty in the zooplankton-phytoplankton relationship.

Zooplankton biomass was weakly negatively correlated with algal diversity, and it is possible that counteractive effects of algal diversity can be manifested through improved chances of balanced nutrition vs. dilution of the most nutritious taxa [13]. This effect sign was the opposite of the one we expected based on prior studies [12,13] possibly because pelagic Great Lakes do not include highly eutrophic waters, where extreme cyanobacterial dominance (and therefore decreased overall algal diversity) is more likely to reduce availability and diversity of preferred algal resources to the extent detrimental to consumers. Zooplankton and phytoplankton Shannon diversity were not significantly correlated in our study, providing additional evidence for inconsistent vertical diversity effects across aquatic ecosystems. Positive vertical diversity effects ~~were~~ observed between bacterial and nanoflaggelate assemblages [55]; however, zooplankton diversity was not predicted by phytoplankton diversity across a wide range of marine systems [56], tropical streams [57], or temperate lakes [58].

We observed stronger correlations between the different zooplankton groups than between zooplankton and phytoplankton. This may indirectly suggest a lack of strong feeding selectivity for zooplankton feeding on phytoplankton, at least at the division level, as well as a lack of general avoidance by zooplankton of Cyanobacteria [38], ability to adapt [59], or a dilution of this pattern by other confounding factors. One of those factors could be availability of picoplankton, which could make an important contribution to the diets of smaller zooplankton. The predator-prey ratio of the zooplankton assemblage was weakly positively predicted by algal diversity, providing marginal support for our hypothesis that more diverse algal assemblages may support greater predator densities.

Understanding the relationships between phytoplankton and zooplankton is important for predicting the effects of changing climate and nutrient loading on food web structure and higher trophic

level [51]. A close correspondence between primary producer and consumer assemblages, indicative of bottom-up regulation, can make consumer populations more vulnerable to changing algal phenology and decreased overall lake productivity. However, we did not observe a close correspondence in the Great Lakes making it more difficult to predict how the higher trophic levels would be affected by the continued changes in phytoplankton assemblages.

References

1. Kosten S, et al. (2012) Warmer climates boost cyanobacterial dominance in shallow lakes. Global Change Biology 18: 118–126.
2. O’Reilly CM, et al. (2015) Rapid and highly variable warming of lake surface waters around the globe. Geophysical Research Letters 42: 10773–10781.
3. Ho JC, Michalak AM, Pahlevan N (2019) Widespread global increase in intense lake phytoplankton blooms since the 1980s. Nature 574: 667–670.
4. Henson SA, Cole HS, Hopkins J, Martin AP, Yool A (2017) Detection of climate-driven trends in phytoplankton phenology. Global Change Biology 24: e101–e111.
5. O’Beirne MD, Werne JP, Hecky RE, Johnson TC, Katsev S, Reavie EE (2017) Anthropogenic climate change has altered primary productivity in Lake Superior. Nature Communications 8: 15713.
6. Rantala MV, et al. (2022) Neoglacial lake‑ ecosystem changes above and below the subarctic Fennoscandian treeline inferred from changes in diatom functional groups. Journal of Paleolimnology, doi.org/10.1007/s10933-022-00272-7
7. T-Krasznai E, et al. (2022) Functional dynamics of phytoplankton assemblages in hypertrophic lakes: Functional- and species diversity is highly resistant to cyanobacterial blooms. Ecological Indicators 145: 109583.

8. Visser ME, Both C (2005) Shifts in phenology due to global climate change: the need for a yardstick. Proceedings of the Royal Society B 272: 2561–2569
9. Durant JM, Hjermann DØ, Ottersen G, Stenseth NC (2007) Climate and the match or mismatch between predator requirements and resource availability. Climate Research 33: 271–283.
10. Asch RG, Stock CA, Sarmiento JL (2019) Climate change impacts on mismatches between phytoplankton blooms and fish spawning phenology. Global Change Biology 25: 2544 – 2559.
11. Ptacnik R, et al. (2008) Diversity predicts stability and resource use efficiency in natural phytoplankton communities. Proceedings of the National Academy of Sciences 105: 5134–5138.
12. Striebel M, Singer G, Stibor H, Andersen T (2012) “Trophic overyielding”: Phytoplankton diversity promotes zooplankton productivity. Ecology 93: 2719–2727.
13. Marzetz V, Koussoroplis AM, Martin-Creuzburg D, Striebel M, Wacker A (2017) Linking primary producer diversity and food quality effects on herbivores: A biochemical perspective. Scientific Reports 7: 11035.
14. López-Rojo N, Boyero L, Pérez J, Basaguren A, Cardinale BJ (2022) No evidence of biodiversity effects on stream ecosystem functioning across green and brown food web pathways. Freshwater Biology 67: 720–730.
15. Filstrup CT, Hillebrand H, Heathcote AJ, Harpole WS, Downing JA (2014) Cyanobacteria dominance influences resource use efficiency and community turnover in phytoplankton and zooplankton communities. Ecology Letters 17: 464–474.
16. Heathcote AJ, Filstrup CT, Kendall D, Downing JA (2016) Biomass pyramids in lake plankton: influence of Cyanobacteria size and abundance. Inland Waters 6: 250 – 257.
17. Wilson AE, Sarnelle O, Tillmanns AR (2006) Effects of cyanobacterial toxicity and morphology on the population growth of freshwater zooplankton: meta-analysis of laboratory experiments. Limnology and Oceanography 51: 1915–1924.

18. Bednarska A, Piertrazak B, Pijanowska J (2014) Effect of poor manageability and low nutritional value of cyanobacteria on Daphnia magna life history performance. Journal of Plankton Research 36: 838–847.
19. Briland RD, Stone JP, Manubolu M, Lee J, Ludsin SA (2020) Cyanobacterial blooms modify food web structure and interactions in western Lake Erie. Harmful Algae 92: 101586.
20. Sterner R, et al. (2017) Grand challenges for research in the Laurentian Great Lakes. Limnology and Oceanography 62: 2510–2523.
21. Ives JT, McMeans BC, McCann KS, Fisk AT, Johnson TB, Bunnell DB, Frank KT, Muir AM (2019) Food-web structure and ecosystem function in the Laurentian Great Lakes—Toward a conceptual model. Freshwater Biology 64: 1–23.
22. Reavie ED, Barbiero RP, Allinger LE, Warren GJ (2014) Phytoplankton trends in the Great Lakes, 2001–2011. Journal of Great Lakes Research 40: 618–639.
23. Reavie ED, Sgro GV, Estepp LR, Bramburger AJ, Shaw Chraïbi VL, Pillsbury RW, Cai M, Stow CA, Dove A (2017) Climate warming and changes in *Cyclotella sensu lato* in the Laurentian Great Lakes. Limnology and Oceanography 62: 768–783.
24. Bramburger AJ, Reavie ED, Sgro GV, Estepp LR, Shaw Chraïbi VL, Pillsbury RW (2017) Decreases in diatom cell size during the 20th century in the Laurentian Great Lakes: a response to warming waters? Journal of Plankton Research 39: 199–210.
25. Barbiero RP, Rudstam LG, Watkins JM, Lesht BM (2019) A cross-lake comparison of crustacean zooplankton communities in the Laurentian Great Lakes, 1997–2016. Journal of Great Lakes Research 45: 672–690.
26. Rudstam LG, Holeck KT, Bowen KL, Watkins JM, Weidel BC, Luckey FJ (2015) Lake Ontario zooplankton in 2003 and 2008: community changes and vertical redistribution. Aquatic Ecosystem Health and Management 18: 43–62.

27. Bourdeau PE, Pangle KL, Peacor SD (2015) Factors affecting the vertical distribution of the zooplankton assemblage in Lake Michigan: the role of the invasive predator *Bythotrephes longimanus*. Journal of Great Lakes Research 41: 415–124.
28. Barbiero RP, Balcer M, Rockwell DC, Tuchman ML (2009) Recent shifts in the crustacean zooplankton community of Lake Huron. Canadian Journal of Fisheries and Aquatic Sciences 66: 816–828.
29. Barbiero RP, Lesht BM, Warren GJ, Rudstam LR, Watkins JM, Reavie ED, Kovalenko KE, Karatayev, AY (2018) A comparative examination of recent changes in nutrients and lower food web structure in Lake Michigan and Lake Huron. Journal of Great Lakes Research 44: 573–589.
30. Kovalenko KE, Reavie ED, Barbiero RP, Burlakova LE, Karatayev AY, Rudstam LG, Watkins JM (2018) Patterns of long-term dynamics of aquatic communities and water quality parameters in the Great Lakes: Are they synchronized? Journal of Great Lakes Research 44: 660–669.
31. Wetzel RG (2001) Limnology: Lake and River Ecosystems. 3rd ed. Academic Press, San Diego. 1024 pp.
32. De Stasio BT, Beranek AE, Schrimpf MB (2018). Zooplankton-phytoplankton interactions in Green Bay, Lake Michigan: Lower food web responses to biological invasions. Journal of Great Lakes Research 44: 910–923.
33. US EPA (2022) Standard Operating Procedure. Great Lakes National Program Office, U.S. Environmental Protection Agency, Chicago, IL.
34. US EPA (2010) SOP LG401, Standard Operating Procedure for Phytoplankton Analysis. Revision 05, February 2010. Great Lakes National Program Office, U.S. Environmental Protection Agency, Chicago, IL.
35. Utermöhl H (1958) Zur vervollkommnung der quantitativen phytoplankton-methodik. Mitteilungen Internationale Vereinigung für Theoretische und Angewandte Limnologie 9: 1–38.

36. Reavie ED, Jicha TM, Angradi TR, Bolgrien DW, Hill BH (2010) Algal assemblages for large river monitoring: Comparison among biovolume, absolute and relative abundance metrics. Ecological Indicators 10: 167–177.
37. McCauley E, Briand F (1979) Zooplankton grazing and phytoplankton species richness: Field tests of the predation hypothesis. Limnology and Oceanography 24: 243–252.
38. Sarnelle O, Gustafsson S, Hansson L-A (2010) Effects of cyanobacteria on fitness components of the herbivore *Daphnia*. Journal of Plankton Research 32: 471–477.
39. Tanković MS, et al. (2018) Experimental evidence for shaping and bloom inducing effects of decapod larvae of *Xantho poressa* (Olivi, 1792) on marine phytoplankton. Journal of the Marine Biological Association of the United Kingdom 98: 1881–1887.
40. US EPA (2017). SOP LG402. Standard Operating Procedure for Zooplankton Sample Collection and Preservation and Secchi Depth Measurement Field Procedures. Revision 12, February 2017. Great Lakes National Program Office, U.S. Environmental Protection Agency, Chicago, IL.
41. US EPA (2017) SOP LG403, Standard Operating Procedure for Zooplankton Analysis. Revision 08, February 2017. Great Lakes National Program Office, U.S. Environmental Protection Agency, Chicago, IL.
42. Nasworthy KC, Scofield AE, Rudstam LG (2020) Feeding ecology of *Limnocalanus macrurus* in the Laurentian Great Lakes. Journal of Great Lakes Research 46: 891–898.
43. R Core Team (2022) R: A language and environment for statistical computing. R Foundation for statistical Computing, Vienna, Austria. https://www.R-project.org/.
44. Bernát G, Boross N, Somogyi B, Vörös L, G.-Tóth L, Boros G (2020) Oligotrophication of Lake Balaton over a 20-year period and its implications for the relationship between phytoplankton and zooplankton biomass. Hydrobiologia 847: 3999–4013.

45. Straile D, Geller W (1998) Crustacean zooplankton in Lake Constance from 1920 to 1995: Response to eutrophication and re-oligotrophication. Advances in Limnology 53: 255–274.
46. Evans TM, et al. (2022) Size spectra analysis of a decade of Laurentian Great Lakes data. Canadian Journal of Fisheries and Aquatic Sciences 79: 183–194.
47. Watson SB, McCauley E, Downing JA (1997) Patterns in phytoplankton taxonomic composition across temperate lakes of differing nutrient status. Limnology and Oceanography 42: 487–495.
48. Downing JA, Watson SB, McCauley E (2010) Predicting Cyanobacteria dominance in lakes. Canadian Journal of Fisheries and Aquatic Sciences 58: 1905–1908.
49. Reinl KL, et al. (2021) Cyanobacterial blooms in oligotrophic lakes: Shifting the high nutrient paradigm. Freshwater Biology 66: 1846–1859.
50. Filstrup CT, Heathcote AJ, Kendall D, Downing JA (2016) Phytoplankton taxonomic compositional shifts across nutrient and light gradients in temperate lakes Inland Waters 6: 234–249.
51. Bunnell DB, et al. (2014) Changing ecosystem dynamics in the Laurentian Great Lakes: bottom-up and top-down regulation. BioScience 64: 26–39.
52. Pothoven SA, Vanderploeg HA (2022) Variable changes in zooplankton phenology associated with the disappearance of the spring phytoplankton bloom in Lake Michigan. Freshwater Bioloby 67: 365–377.
53. Lehman JT, Caceres CE (1993) Food-web responses to species invasion by a predatory invertebrate - Bythotrephes in Lake Michigan. Limnology and Oceanography 38: 879–891.
54. Pangle KL, Peacor SD, Johannsson OE (2007) Large nonlethal effects of an invasive invertebrate predator on zooplankton population growth rate. Ecology 88: 402–412.
55. Yang JW, et al. (2018) Predator and prey biodiversity relationship and its consequences on marine ecosystem functioning—interplay between nanoflagellates and bacterioplankton. The ISME Journal 12: 1532–1542.

56. Irigoien X, Huisman J, Harris RP (2004) Global biodiversity patterns of marine phytoplankton and zooplankton. Nature 429: 863–867.
57. Faquim RCP, et al. (2021) Shortcuts for biomonitoring programs of stream ecosystems: Evaluating the taxonomic, numeric, and cross-taxa congruence in phytoplankton, periphyton, zooplankton, and fish assemblages. PLoS ONE 16: e0258342.
58. Allen AP, et al. (1999) Concordance of taxonomic richness patterns across multiple assemblages in lakes of the northeastern United States. Canadian Journal of Fisheries and Aquatic Sciences 56: 739–747.
59. Schaffner L, et al. (2019) Consumer-resource dynamics is an eco-evolutionary process in a natural plankton community. Nature Ecology and Evolution 3: 1251–1258.

Figure 1

Click here to access/download;Figure;fig1_phytoZoo

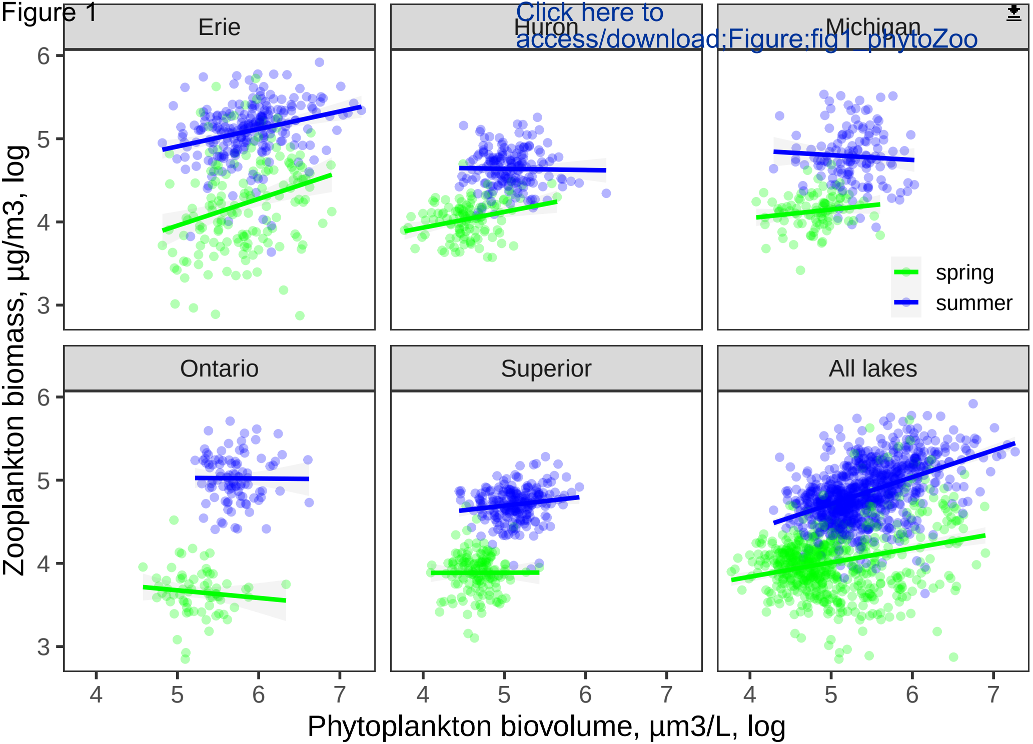

Figure 2

Click here to access/download;Figure;fig2_phytoZooBiov_yr.pdf

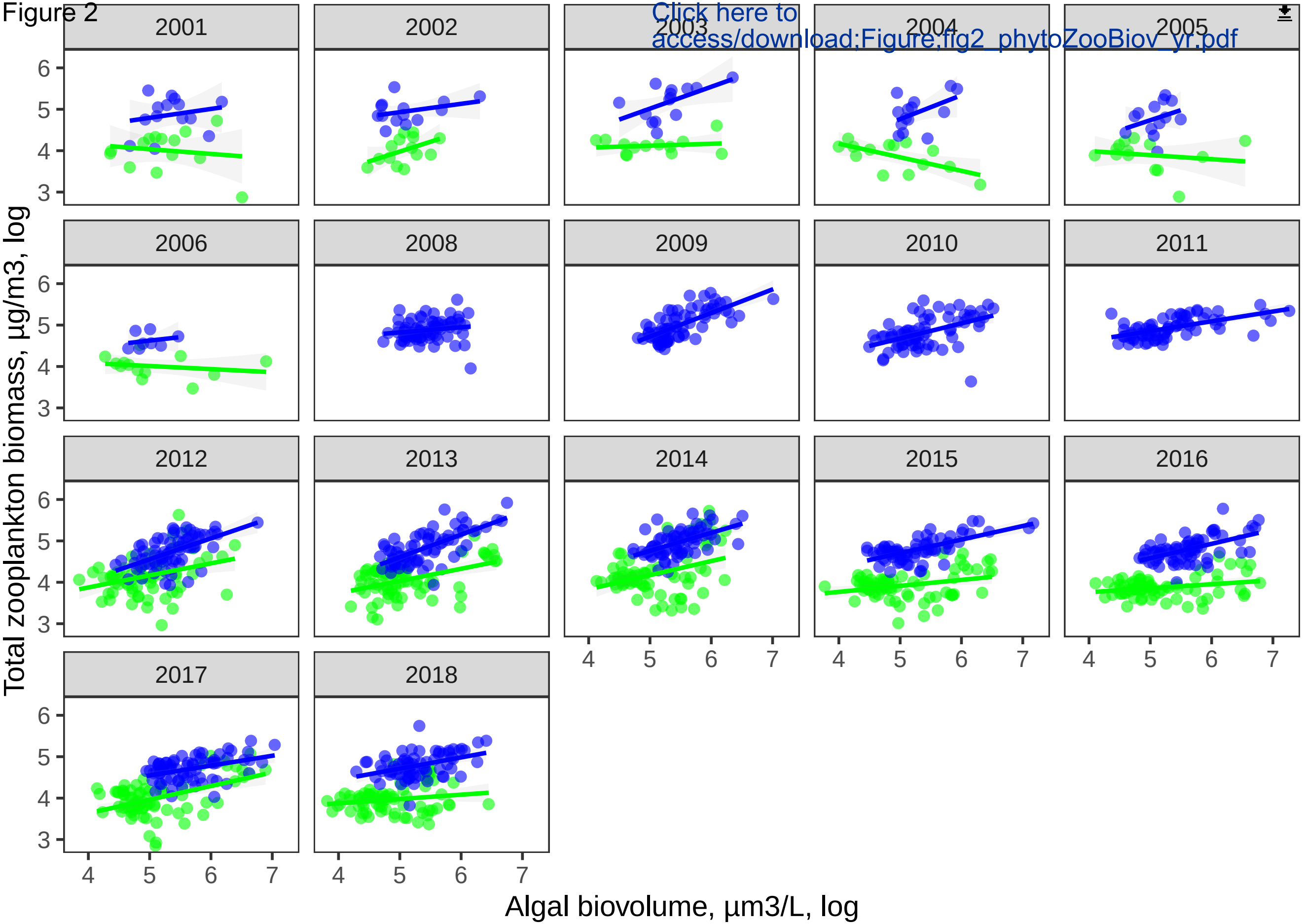

Figure 3

Click here to access/download;Figure;fig3_phytoHZo

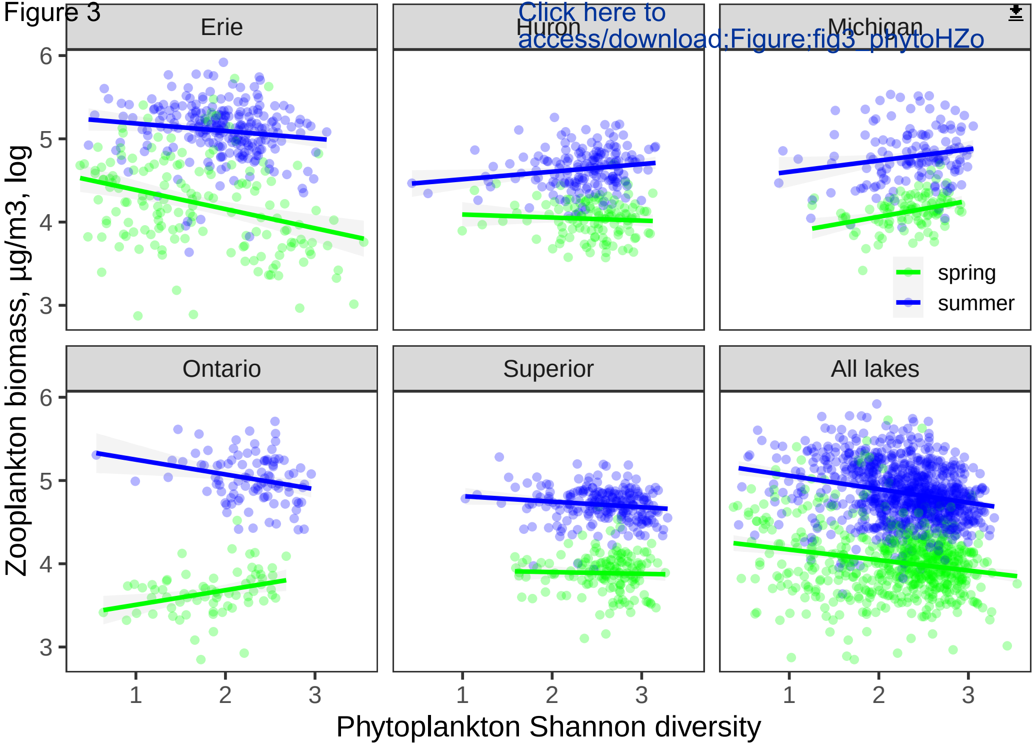

Figure 4

Click here to access/download;Figure;fig4_crossCorr_Jan

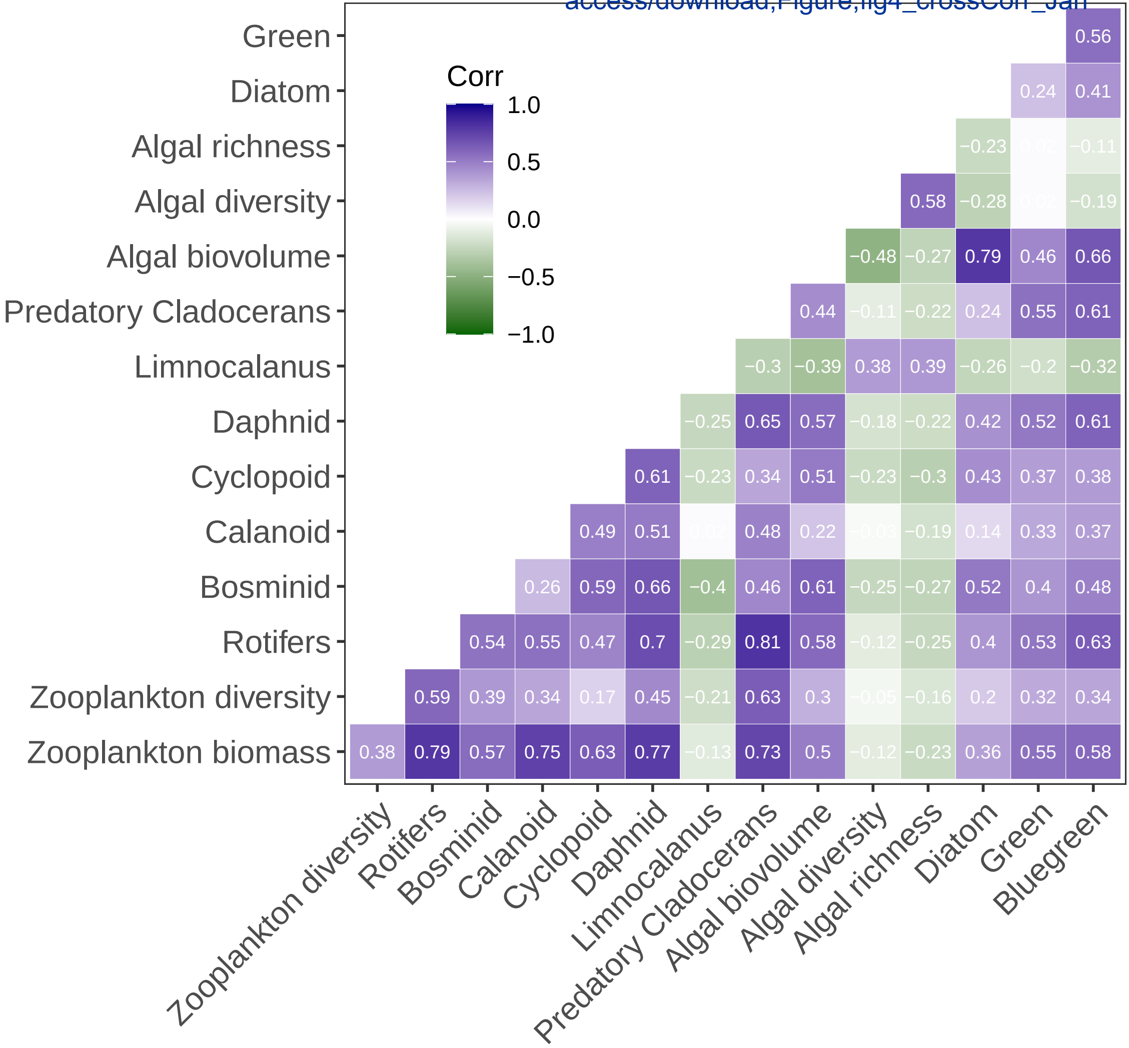

Figure 5

Click here to access/download;Figure;fig5_alBiovEdib

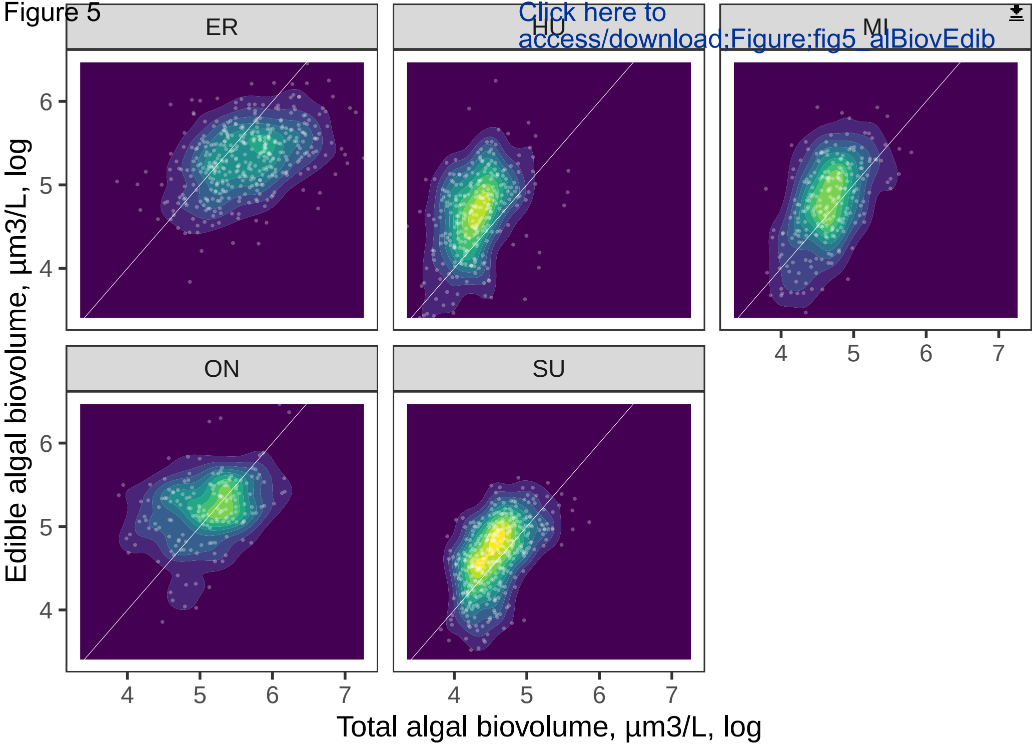

Click here to access/download
Supporting Information
Supplemental Figures.docx

SI Table S1

Click here to access/download
Supporting Information
tableS1.csv

SI Table S2

Click here to access/download
Supporting Information
tableS2.csv
