# Supplementary material for: Zooplankton-phytoplankton biomass and diversity relationships in the Great Lakes
Source: PLoS One. 2023 Oct 26;18(10):e0292988. doi: 10.1371/journal.pone.0292988 (PMC10602272; doi:10.1371/journal.pone.0292988)
Supplement: Attachment — Submitted filename: responsesReviewCommentsKovalenko2.docx [file pone.0292988.s013.docx]

PONE-D-23-03077 Zooplankton-phytoplankton biomass and diversity relationships in the Great Lakes

Dear Dr. Kovalenko,

Thank you for submitting your manuscript to PLOS ONE. After careful consideration, we feel that it has merit but does not fully meet PLOS ONE’s publication criteria as it currently stands. Therefore, we invite you to submit a revised version of the manuscript that addresses the points raised during the review process.

Two reviewers see merit in the manuscript, but both suggest major revision. Reviewer 1 emphasizes a lack of detail in the methods, and the need to temper some statements in the light of tenuous correlations. This reviewer provided a marked up copy of the manuscript with comments and edits. I urge you to look carefully at that document.

The second reviewer is concerned that you are missing recent relevant literature and that your discussion misses some mechanistic explanations.

Please, carefully consider both reviewers' comments and suggestions if you decide to submit a revised version of the manuscript.

Please submit your revised manuscript by May 26 2023 11:59PM. If you will need more time than this to complete your revisions, please reply to this message or contact the journal office at plosone@plos.org.

Please include the following items when submitting your revised manuscript:

A rebuttal letter that responds to each point raised by the academic editor and reviewer(s). You should upload this letter as a separate file labeled 'Response to Reviewers'.

A marked-up copy of your manuscript that highlights changes made to the original version. You should upload this as a separate file labeled 'Revised Manuscript with Track Changes'.

An unmarked version of your revised paper without tracked changes. You should upload this as a separate file labeled 'Manuscript'.

If you would like to make changes to your financial disclosure, please include your updated statement in your cover letter. Guidelines for resubmitting your figure files are available below the reviewer comments at the end of this letter.

If applicable, we recommend that you deposit your laboratory protocols in protocols.io to enhance the reproducibility of your results. Protocols.io assigns your protocol its own identifier (DOI) so that it can be cited independently in the future. For instructions see: https://journals.plos.org/plosone/s/submission-guidelines#loc-laboratory-protocols. Additionally, PLOS ONE offers an option for publishing peer-reviewed Lab Protocol articles, which describe protocols hosted on protocols.io. Read more information on sharing protocols at https://plos.org/protocols?utm_medium=editorial-email&utm_source=authorletters&utm_campaign=protocols.

We look forward to receiving your revised manuscript.

Kind regards,

Hans G. Dam, Ph. D.

Academic Editor

PLOS ONE

______Dear Dr. Dam,

Thank you for your editorial work on this manuscript. We have addressed all reviewer comments as detailed below. To summarize those changes, we have expanded the discussion with more relevant literature and mechanistic explanations and tempered all parts of the ms to reflect the very low ecological effect sizes (R2). Please note that we cite new references using names in the response letter for an easier evaluation, these are cited numerically in text.

Please note the updated financial disclosure and a statement about the role of the funding agency: the US Environmental Protection Agency is the key entity collecting these data, and, as such, it is involved in study design and sampling, it would not have been possible for individual researchers to conduct sampling at these scales. I apologize for not clearly stating that in the disclosure.

Sincerely, katya, on behalf of other co-authors.

Journal requirements:

When submitting your revision, we need you to address these additional requirements.

1. Please ensure that your manuscript meets PLOS ONE's style requirements, including those for file naming. The PLOS ONE style templates can be found at

https://journals.plos.org/plosone/s/file?id=wjVg/PLOSOne_formatting_sample_main_body.pdf and

<https://journals.plos.org/plosone/s/file?id=ba62/PLOSOne_formatting_sample_title_authors_affiliations.pdf>

______All figure and table files have been renamed as per requirements.

2. We note that you have stated that you will provide repository information for your data at acceptance. Should your manuscript be accepted for publication, we will hold it until you provide the relevant accession numbers or DOIs necessary to access your data. If you wish to make changes to your Data Availability statement, please describe these changes in your cover letter and we will update your Data Availability statement to reflect the information you provide.

______We will make all data available via Environmental Protection Agency’s Environmental Dataset Gateway (https://edg.epa.gov).

3. Please include captions for your Supporting Information files at the end of your manuscript, and update any in-text citations to match accordingly. Please see our Supporting Information guidelines for more information: <http://journals.plos.org/plosone/s/supporting-information>.

______SI captions have been added according to the guidelines.

[Note: HTML markup is below. Please do not edit.]

**Reviewers' comments:**

Reviewer's Responses to Questions

Comments to the Author

1. Is the manuscript technically sound, and do the data support the conclusions?

The manuscript must describe a technically sound piece of scientific research with data that supports the conclusions. Experiments must have been conducted rigorously, with appropriate controls, replication, and sample sizes. The conclusions must be drawn appropriately based on the data presented.

Reviewer #1: Yes

Reviewer #2: Yes

2. Has the statistical analysis been performed appropriately and rigorously?

Reviewer #1: Yes

Reviewer #2: Yes

3. Have the authors made all data underlying the findings in their manuscript fully available?

The PLOS Data policy requires authors to make all data underlying the findings described in their manuscript fully available without restriction, with rare exception (please refer to the Data Availability Statement in the manuscript PDF file). The data should be provided as part of the manuscript or its supporting information, or deposited to a public repository. For example, in addition to summary statistics, the data points behind means, medians and variance measures should be available. If there are restrictions on publicly sharing data—e.g. participant privacy or use of data from a third party—those must be specified.

Reviewer #1: Yes

Reviewer #2: Yes

4. Is the manuscript presented in an intelligible fashion and written in standard English?

PLOS ONE does not copyedit accepted manuscripts, so the language in submitted articles must be clear, correct, and unambiguous. Any typographical or grammatical errors should be corrected at revision, so please note any specific errors here.

Reviewer #1: Yes

Reviewer #2: Yes

5. Review Comments to the Author

Please use the space provided to explain your answers to the questions above. You may also include additional comments for the author, including concerns about dual publication, research ethics, or publication ethics. (Please upload your review as an attachment if it exceeds 20,000 characters)

**Reviewer #1:**

• The weak correlations presented here should be treated as such and conclusions based on these relationships should be considered with caution to not over emphasize their significance.

______Abstract, results, and discussion were closely reviewed to ensure that the significance of these weak trends has not been over-emphasized. A sentence in the abstract was modified from “limited evidence” to “no evidence”; another sentence in results referring to zooplankton biomass-phytoplankton diversity relationship was changed to emphasize how weak the relationship was; the first sentence of Discussion was also abated to highlight that as well “There was a statistically significant but weak correlation”. We made several other edits to ensure that the text clearly refers to “weak correlations” throughout. In addition, we have added a more detailed discussion of limitations as was suggested below.

• The paper is well written, but limited in the details included in the methods section. Additional details should be included, particularly in the statistical analysis section as how the analysis was accomplished (Time to process, diversity index, spearman correlation, etc)

______We have added time of day, Spearman index, Shannon diversity and other missing analyses (including GAMs and time of day) to the methods. (We think “time to process” in the comment above refers to time of day, because it is mentioned in the context of statistical analysis.)

• Some limitations of the data were brought up (edibility), however all limitations (integrated samples, net speed and size, day/night, only up to 2 sample points per station per year, minimum of 2 stations per lake) need to be discussed and the implications considered.

______We have now added discussion of additional limitations as requested: “It is important to note that over these time scales, our dataset has temporal sampling limitations (only 2 sampling events/station/year) and lower number of stations sampled in the earlier years. Integrated samples are collected from the isothermal upper layer of the water column to favor even sampling of the phytoplankton assemblage. Although we did not see time of sampling explaining additional variation, other studies have shown that many zooplankton species have pronounced vertical migration (Watkins et al. 2017, Doubek et al. 2020, Scofield et al. 2020) which could further contribute to the observed uncertainty about zooplankton-phytoplankton relationships. All of these factors may limit our ability to draw conclusions about the strength of temporal trends across the entire study period.”

• Several figure and table captions contained methods or analysis results not discussed in the body of the paper. The authors should review all captions to ensure that all appropriate content is included in the body of the text.

______We checked all legends and captions for the missing information, and we added the following information to methods: a) and fewer stations had matching data for the two assemblages earlier in the time series; b) [diversity] (Shannon H); c) [log-transformation was applied to reduce skewness when warranted](biovolume and biomass data); d) Spearman [cross-correlation]; e) time of day analyses; and f) Generalized Additive Models and model parameters.

• Additional comments and edit suggestions made directly in the attached PDF.

______All edits addressed as follows: Use of time of day and inferring vertical migration should be noted in the methods

______Added.

Fig 2S. What does the blue line indicate?

______Added “Generalized Additive Model (GAM) fit” and appropriate details in Methods.

move "demonstrating relatively minor effects of time of day despite pronounced vertical migration in some zooplankton taxa." to results.

______Moved to results, rephrasing the statement in the results as “Examining zooplankton-phytoplankton relationship by the time of sampling demonstrated relatively minor effects of time of day on the shape of the biovolume-biomass relationship in individual lakes”.

move "demonstrating lack of a univariate relationship (e.g., decreasing or increasing proportion of edible biovolume)." to results.

______This statement, slightly rephrased is already in the Results, therefore it was simply omitted from the legend to avoid repetition.

**Reviewer #2:** Dear Author,

your study based on the ratio of predator-to-prey biomass as a key element of trophic structure of lake ecosystems. It is typically investigated from a food chain perspective, ignoring channels of energy transfer that may govern community structure where you study try to approach those processes across space and time.

Line 42-43: I would suggest to add some important recent studies on the predator-prey biomass ratio that have shown that can be stable. In general I would suggest to ass more literature on paper that have been testing this ration and explain what they have been observing.

literature example: Perkins et al. 2022 Consistent predator-prey biomass scaling in complex food webs.

______Thank you for suggesting this key reference, it has now been cited, along with Hatton et al. 2015. We also added a reference to predator-prey biomass ratio declining in response to stress (drought) in another system (Ruiz et al. 2022) and expanded the overview of potential drivers in the Discussion. Specifically, we added: “Predator-prey biomass ratios can respond to environmental stressors because predators can take longer to recover from perturbations, at least in isolated environments (Ruiz et al. 2022); however, other studies show remarkable consistency in predator-prey ratios across a wide range of taxa and systems (Hatton et al. 2015, Perkins et al. 2022).”

Line 50-53: I think there are studies that show changes of size spectra, as well as biomass across time and space. I would suggest to include those studies because there are closely related with what you are testing in this study.

______Added: “Decreasing algal cell sizes in particular [24] could have repercussions for the entire aquatic food web, consistent with a climate change signal linked to decreasing community, species and individual sizes across a range of aquatic organisms and systems (Daufresne et al. 2009). However, in the Great Lakes, size spectra slopes, indicative of energy transfer efficiency, remained relatively stable over time (Evans et al. 2022).”

Line 195-196: How do you explain this? This a pattern that have been observed or not by other studies you should include them and try to explain why you studies confirm on not what other paper have found. You describe the patterns but you haven't tried to explain them.

______Added more detailed discussion with many potential mechanisms: “The match/mismatch hypothesis focuses on the consequences of inter-specific differences in response to climate change leading to potentially non-linear responses in the patterns of synchrony [9]. Such decoupling has already been observed in other systems as a result of a mismatch between trophic levels responding primarily to photoperiod vs. those responding to temperature [53]. In the Great Lakes, decreasing diatom cell sizes due to accelerated loss of larger individuals during summer stratification [24], for example, could make consumers rely on less energetically optimal smaller-sized algae. Longer ice-free periods in Lake Superior have resulted in longer stratification and increased primary production [5] and could lead to a timing mismatch between the peak of the spring bloom and zooplankton reproduction.”

Line 198-199: I don't think that your have tested the size spectra, maybe you mean something else. Please explain.

______That is correct, we did not test the size spectra but other study did with these data, which we cite as a potential explanation for the lack of changes in coupling (i.e., if assemblage composition changed but size spectra remained similar, it may be not as noticeable to the consumers). Unless we misunderstood the comment, no change is necessary, but we would be glad to revisit this if needed.

Line 208-210: Are all the zooplankton species included in the study herbivores. If not then any you have to separate them cause the carnivore zooplankton is most probably not going to respond on the phytoplankton biovolume changes. Also the presence of carnivore zooplankton may affect the size of the herbivore zooplankton that may in turn be affected by the phytoplankton volume. so it may be a combined affect of those two. This is maybe something to include in the discussion session.

______We have now repeated biomass analyses with the carnivorous zooplankton excluded. This made the greatest difference in total biomass in Lake Superior; however, it did not affect the strength of zooplankton biomass-phytoplankton biovolume relationship; or biomass-phytoplankton diversity relationship. As such, we have added those figures to Supporting Information, and are discussing them in the results. We are discussing the top-down effects of invertebrate and fish predation on ln. 242-244.

I think in general the discussion session is missing important literature about their field as well as some possible mechanistic explanation about the patterns you are observing. But the pattern you observe are interesting!

______We thank the reviewer for this assessment, and we have now expanded discussion to include potential mechanistic explanations of size spectra, match/mismatch hypothesis and predator-prey ratios as detailed above.

6. PLOS authors have the option to publish the peer review history of their article (what does this mean?). If published, this will include your full peer review and any attached files.

If you choose “no”, your identity will remain anonymous but your review may still be made public.

Do you want your identity to be public for this peer review? For information about this choice, including consent withdrawal, please see our Privacy Policy.

Reviewer #1: No

Reviewer #2: No
