# Supplementary material for: Zooplankton-phytoplankton biomass and diversity relationships in the Great Lakes
Source: PLoS One. 2023 Oct 26;18(10):e0292988. doi: 10.1371/journal.pone.0292988 (PMC10602272; doi:10.1371/journal.pone.0292988)
Supplement: Attachment — Submitted filename: responsesReviewCommentsKovalenkoRound2.docx [file pone.0292988.s014.docx]

PONE-D-23-03077R1

Zooplankton-phytoplankton biomass and diversity relationships in the Great Lakes

PLOS ONE

Dear Dr. Kovalenko,

I have now received feedback from the two original reviewers of your manuscript on the revised version. One reviewer stated directly to me: " The revised manuscript addresses all the comments/suggestions made by the reviewers and I'm very pleased that you have extended your analysis. The present revised manuscript makes an excellent an timing contribution to PLOS ONE." The other review is copied below, and you can see that it contains stylistic suggestions and comments to improve the final version of the paper. Once you make those, i can move to accept the paper. Like the reviewer, I was unable to open the attached figures in the manuscript. Please ensure they are in a format that the journal accepts.

Please submit your revised manuscript by Sep 29 2023 11:59PM. If you will need more time than this to complete your revisions, please reply to this message or contact the journal office at plosone@plos.org. When you're ready to submit your revision, log on to https://www.editorialmanager.com/pone/ and select the 'Submissions Needing Revision' folder to locate your manuscript file.

Please include the following items when submitting your revised manuscript:

A rebuttal letter that responds to each point raised by the academic editor and reviewer(s). You should upload this letter as a separate file labeled 'Response to Reviewers'.

A marked-up copy of your manuscript that highlights changes made to the original version. You should upload this as a separate file labeled 'Revised Manuscript with Track Changes'.

An unmarked version of your revised paper without tracked changes. You should upload this as a separate file labeled 'Manuscript'.

If you would like to make changes to your financial disclosure, please include your updated statement in your cover letter. Guidelines for resubmitting your figure files are available below the reviewer comments at the end of this letter.

If applicable, we recommend that you deposit your laboratory protocols in protocols.io to enhance the reproducibility of your results. Protocols.io assigns your protocol its own identifier (DOI) so that it can be cited independently in the future. For instructions see: https://journals.plos.org/plosone/s/submission-guidelines#loc-laboratory-protocols. Additionally, PLOS ONE offers an option for publishing peer-reviewed Lab Protocol articles, which describe protocols hosted on protocols.io. Read more information on sharing protocols at https://plos.org/protocols?utm_medium=editorial-email&utm_source=authorletters&utm_campaign=protocols.

We look forward to receiving your revised manuscript.

Kind regards,

Hans G. Dam, Ph. D.

Academic Editor

PLOS ONE

Journal Requirements:

Please review your reference list to ensure that it is complete and correct. If you have cited papers that have been retracted, please include the rationale for doing so in the manuscript text, or remove these references and replace them with relevant current references. Any changes to the reference list should be mentioned in the rebuttal letter that accompanies your revised manuscript. If you need to cite a retracted article, indicate the article’s retracted status in the References list and also include a citation and full reference for the retraction notice.

[Note: HTML markup is below. Please do not edit.]

Reviewers' comments:

Reviewer's Responses to Questions

Comments to the Author

1. If the authors have adequately addressed your comments raised in a previous round of review and you feel that this manuscript is now acceptable for publication, you may indicate that here to bypass the “Comments to the Author” section, enter your conflict of interest statement in the “Confidential to Editor” section, and submit your "Accept" recommendation.

Reviewer #1: (No Response)

2. Is the manuscript technically sound, and do the data support the conclusions?

The manuscript must describe a technically sound piece of scientific research with data that supports the conclusions. Experiments must have been conducted rigorously, with appropriate controls, replication, and sample sizes. The conclusions must be drawn appropriately based on the data presented.

Reviewer #1: Yes

3. Has the statistical analysis been performed appropriately and rigorously?

Reviewer #1: Yes

4. Have the authors made all data underlying the findings in their manuscript fully available?

The PLOS Data policy requires authors to make all data underlying the findings described in their manuscript fully available without restriction, with rare exception (please refer to the Data Availability Statement in the manuscript PDF file). The data should be provided as part of the manuscript or its supporting information, or deposited to a public repository. For example, in addition to summary statistics, the data points behind means, medians and variance measures should be available. If there are restrictions on publicly sharing data—e.g. participant privacy or use of data from a third party—those must be specified.

Reviewer #1: Yes

5. Is the manuscript presented in an intelligible fashion and written in standard English?

PLOS ONE does not copyedit accepted manuscripts, so the language in submitted articles must be clear, correct, and unambiguous. Any typographical or grammatical errors should be corrected at revision, so please note any specific errors here.

Reviewer #1: Yes

6. Review Comments to the Author

Please use the space provided to explain your answers to the questions above. You may also include additional comments for the author, including concerns about dual publication, research ethics, or publication ethics. (Please upload your review as an attachment if it exceeds 20,000 characters)

Reviewer #1: Thank you to the authors for their revisions.

Please see my comments on the following minor concerns:

- Line 12 (and lines 73 and 83), EPA should defined at first use. If not in the abstract then in the introduction.

______ Defined as suggested.

- Lines 48-49, this sentence is awkward. the authors should revise the sentence to avoid the double use of "can" and clarify this statement.

______ Thank you for pointing this out, rephrased as “Predator-prey biomass ratios can respond to environmental stressors when predators take longer to recover from perturbations, e.g., in isolated environments [20]; however, other studies show remarkable consistency in predator-prey ratios across a wide range of taxa and systems [21,22].”

- Lines 200-202 (Figure Caption for Figure 5), the authors should include full lake names in the figures for consistency with the other figures included in the text, (not supplemental), or define the acronyms in the caption.

_______ Done, full lake names included.

- Line 267, what do the authors mean by "dilution of this pattern". Perhaps this can be reworded for clarity.

______ Clarified as “masking of feeding selectivity by other confounding factors”, meaning that we cannot exclude presence of feeding selectivity however it may be masked by other, stronger drivers of phytoplankton abundance.

- Figure 1, the units should be properly formatted as superscript in the figure axes.

_______ Done.

- Figure 2, the units should be properly formatted as superscript in the figure axes.

_______ Done.

- Figure 3, the units should be properly formatted as superscript in the figure axis.

_______ Done.

- Figure 4, The colors for this figure, particularly for Limnocalanus, and algal diversity are difficult to read on a computer screen and near impossible to read when printed. I strongly recommend the authors choose a different text color as the white text on white correlation is bad.

______In this case, we adjusted the color of the text and background specifically so that non-significant correlations (with very low R values) do not show up. Because this was intentional, we have not changed the figure but clarified it better in the legend.>>>

- Figure 5, the units should be properly formatted as superscript in the figure axis. Additionally, the acronyms as described above.

_______ Done.

- FigS2, I can't open this file. Is this file corrupt?

- FigS3, the units should be properly formatted as superscript in the figure axes.

- FigS4, the units should be properly formatted as superscript in the figure axis.

- FigS5, the units should be properly formatted as superscript in the figure axes.

- FigS6, the units should be properly formatted as superscript in the figure axis.

- FigS7, the units should be properly formatted as superscript in the figure axes. Additionally, 'Spr' and 'Sum' do not need to be shortened here, they could easily be written out without impacting the figure.

_______ All supplemental figures have been redone to address reviewer comments. Fig S2 was redone to ensure it is not corrupt. The current version opens fine, but all .eps figures (as required by the journal) need a postscript reader or need to be converted to a .pdf.

7. PLOS authors have the option to publish the peer review history of their article (what does this mean?). If published, this will include your full peer review and any attached files.

If you choose “no”, your identity will remain anonymous but your review may still be made public.

Do you want your identity to be public for this peer review? For information about this choice, including consent withdrawal, please see our Privacy Policy.

Reviewer #1: No

[NOTE: If reviewer comments were submitted as an attachment file, they will be attached to this email and accessible via the submission site. Please log into your account, locate the manuscript record, and check for the action link "View Attachments". If this link does not appear, there are no attachment files.]

While revising your submission, please upload your figure files to the Preflight Analysis and Conversion Engine (PACE) digital diagnostic tool, https://pacev2.apexcovantage.com/. PACE helps ensure that figures meet PLOS requirements. To use PACE, you must first register as a user. Registration is free. Then, login and navigate to the UPLOAD tab, where you will find detailed instructions on how to use the tool. If you encounter any issues or have any questions when using PACE, please email PLOS at figures@plos.org. Please note that Supporting Information files do not need this step.

In compliance with data protection regulations, you may request that we remove your personal registration details at any time. (Remove my information/details). Please contact the publication office if you have any questions.
